# Position paper: ability to drive in cerebrovascular diseases

**DOI:** 10.1186/s42466-019-0043-z

**Published:** 2019-10-23

**Authors:** Peter Marx, Gerhard Hamann, Otto Busse, Thomas Mokrusch, Hendrik Niemann, Hartmut Vatter, Bernhard Widder

**Affiliations:** 1 Department of Neurology, Campus Benjamin Franklin, Charitè Universtitätsmedizin Berlin, Terrassenstr. 45, 14129 Berlin, Germany; 2 Department of Neurology, Bezirkskrankenhaus Günzburg, Günzburg, Germany; 3 Deutsche Schlaganfallgesellschaft, Berlin, Germany; 4 Department of Neurological Rehabilitation, MediClin Hedon Klinik Lingen, Lingen, Germany; 5 NRZ Center of Neurological Rehabilitation, Leipzig, Germany; 6 Department of Neusosurgery, Friedrich-Wilhelm-University Bonn, Bonn, Germany; 7 Institute of medico-legal Assessment, Bezirkskrankenhaus Günzburg, Günzburg, Germany

**Keywords:** Stroke, Transient ischemic attack, Intracerebral bleeding, Subarachnoid bleeding, Informed consent, Fitness to drive

## Abstract

The regulations for fitness to drive after a cerebrovascular accident in the German Driving License Regulations (FeV) and the German Evaluation Guidelines for Driving Ability (BGL). are not up to date with the current medical knowledge and not consistent with regulations regarding cardiovascular diseases.

This position paper presented by six medical and neuropsychological societies in Germany provides a guideline for the assessment of driving ability after diagnosis of a cerebrovascular disease and addresses three major questions:

If there is a functional limitation, how can it be compensated for?

What is the risk of sudden loss of control while driving in the future?

Are there behavioral or personality changes or cognitive deficiencies interfering with safety while driving?

Recommendations for the assessment of driving ability in different cerebrovascular diseases are presented.

This article is a translation of the position paper published in Nervenarzt: Marx, P., Hamann, G.F., Busse, O. et al. Nervenarzt 90(4): 388–398.

## Introduction

Driving is a vital aspect of many people’s daily lives. German law requires that any driver exhibit good health, with no significant impairment of ability to drive. In addition to epilepsy and cardiovascular diseases, cerebrovascular diseases present a major group of diseases which can negatively impact the ability to drive. The right to drive a motor vehicle is offset by the potential danger entailed, which is why driving ability and driver’s licenses are subject to strict legal conditions. Knowledge of the medical hazard potential and the legal requirements are important for both the physician giving mandatory advice within the scope of safety assessment and the certifying medical expert. The recommendations presented in this position paper are based on the interpretation of clinical studies by experts entrusted with this task by the following societies:

- German Society of Neuroscientific Assessment (DGNB)
- German Society of Neurology (DGN)
- German Society of Neurosurgery (DGNC)
- German Society of Neurorehabilitation (DGNR)
- German Stroke Society (DSG)
- Society for Neuropsychology (GNP)

A complete version of this position paper can be found on the websites of the DGNB, DGNC, DGNR and GNP.

## Legal requirements

According to §2 (4) of the German Road Traffic Act (Straßenverkehrsgesetz, StVG) [1], anyone who fulfills the necessary physical and mental requirements and has not significantly or repeatedly violated traffic regulations or criminal laws is fit for driving motor vehicles.

More detailed provisions are specified in the EU Driving Licence Directive [2], which is the basis for the German Driving Permission Act (Fahrerlaubnisverordnung, FeV) [3] of the Federal Ministry of Justice and Consumer Protection. According to § 11 of the FeV, the requirements for fitness to drive are “not fulfilled if an illness or a defect according to Annex 4 or 5 is present, whereby the fitness or the conditional fitness to drive motor vehicles is excluded”.

Additionally, the German medical-psychological advisory board for traffic medicine of the Bundesanstalt für Straßenwesen (BASt) publishes the Evaluation Guidelines for Driving Ability (BGL) [4]. These guidelines are intended to be used as a decision-making aid for individual cases. Deviating assessments are possible in principle, but require detailed justification.

In Germany, there is no obligation to report illnesses that restrict fitness to drive, nor is a driver’s license automatically withdrawn after a relevant illness is diagnosed. However, the individual concerned is obliged to take precautions to ensure safe participation in road traffic so that others are not endangered “as a result of physical or mental deficiencies”. If an individual drives despite relevant performance restrictions, this can result in loss of driver’s license, loss of insurance coverage and even criminal prosecution. According to § 2 (8) of the StVG, the licensing authority only intervenes when facts become known which give rise to concerns about fitness to drive. Neither the StVG nor the FeV obligate the treating physician to report to the driver licensing authority in the case of an existing illness.

### Annex 4 of the FeV and Annex 4 of the evaluation guidelines for driving ability (BGL)

Driver’s license classes are divided into two groups. Group 1 comprises categories A, A1, A2, B, BE, AM, L and T (motor-cycles, passenger cars, trucks < 3.5 tons); group 2 comprises categories C, C1, CE, C1E, D, D1, DE, D1E (trucks > 3.5 tons, busses) and passenger transport licenses (FzF).

Appendix 4 of the FeV and Annex 4 of the BGL [4] both contain a list of common illnesses and defects which may impair the ability to drive motor vehicles. For cerebrovascular diseases, here referred to as “circulatory disorders of brain activity” (Section 3.9.4 BGL), fitness to drive is only recognized for group 1 after successful therapy and decay of the acute event without risk of relapse, but denied altogether for group 2.

Regarding ability to drive, the following medical questions arise after a cerebrovascular disease is diagnosed:

- Are there any physical or mental functional limitations that permanently impair driving ability? If so, are there any ways of compensating for these functional limitations, such as making driving fitness subject to conditions or restrictions?
- How high is the risk potential of a sudden loss of control as a result of another stroke or cardiovascular event while driving?
- Are there deficiencies in self-control or attitudes contrary to safe behavior?

## Evaluation of physical and mental dysfunctions

Cerebrovascular diseases can cause prolonged neurocognitive, sensory (e.g. proprioception, vision, hearing), and, above all, motor dysfunctions as well as impairments of balance.

**Table:** 

**Recommendation** Patients suffering from any form of cerebrovascular disease with substantial initial disability (modified Rankin Scale, mRS > 2) should be evaluated for their driving fitness during the rehabilitation process. A detailed summary should be included in the final discharge documents.	

### Neurocognitive impairments

In addition to the functional areas mentioned in Section 2.5 of the BGL [4], disorders of learning and memory, visual spatial perception including neglect (see Chapter 3.2), and executive functions (e.g., impulse control, error monitoring, anticipatory planning and problem solving) should also be considered after a cerebrovascular disease is diagnosed.

**Table:** 

**Recommendation** If minimum cognitive requirements, as defined in the German Evaluation Guidelines for Driving Ability (BGL), are not met, but the patient still wishes to drive, an on-road driving test with a neuropsychologist should be recommended to the patient. The same applies in the case of changes to emotional control, awareness, or personality, all of which may result in unsafe driving. In individual cases, risk can be reduced to an acceptable level by, for example, limiting driving to certain vehicle types (e.g., automatic transmission), familiar surroundings, or daytime only.	

### Neglect

Neglect is a multimodal cognitive disorder, as generally more than one modality (e.g., vision, hearing or motor skills) is affected. When driving, visual neglect to the left or right is associated with a high risk of accident. As long as the visual neglect interferes with activities of daily living (e.g., dressing, personal hygiene, eating, and moving about the home) driving a car is not an option. Information provided by observers (e.g., rehabilitation staff or family members) is always necessary, as patient self-reports alone are not sufficient.

**Table:** 

**Recommendation** **Group 1** If the visual neglect without additional visual field loss has improved to such an extent that it can no longer be observed by others (e.g., therapists or family members) an on-road driving test of at least 60 min should be considered. The driving evaluation should be done in such a way that the neglect is specifically evaluated (e.g., in inner city traffic at rush hour). This test should be carried out together with a neuropsychologist. **Group 2** As a rule, safe driving of group 2 vehicles (e.g., trucks) cannot be expected because of the additional demands on cognitive resources (e.g., sustained attention over long periods of time) even if the neglect has improved and cannot be observed by others. In rare cases, a driving evaluation can be carried out with appropriate vehicles and for longer time periods to test the stability of functioning.	

### Language impairments

Studies on fitness to drive with aphasic patients [5, 6] have shown that aphasia as such is not associated with unsafe driving. Aphasic drivers do not differ significantly from healthy controls with regard to the result of a standardized on-road driving test. Only patients with global aphasia were more often unable to drive safely, which may be due to additional neurocognitive impairments.

**Table:** 

**Recommendation** **Group 1** In general, an on-road driving evaluation is recommended for aphasic patients, if possible accompanied by a neuropsychologist. In this context, it should also be assessed whether comprehension of traffic signs is impaired. **Group 2** The on-road driving test should be carried out with an appropriate group 2 vehicle and for longer time periods to test the stability of functioning (minimum driving duration 60 min).	

### Vascular dementia

Vascular dementia with acute onset (F01.0), multi-infarct dementia (F01.1), subcortical vascular dementia (F01.2), and mixed cortical/subcortical vascular dementia (F01.3) all interfere with driving ability. This disease group can present with disorders of orientation, attention, language, visual-spatial abilities, judgement, ability to act, abstraction, motor control or praxis [7]. The combination of vascular dementia with dementia of the Alzheimer type is called mixed dementia.

For the assessment of fitness to drive, both the current extent of the impairments and the risk of progression must be taken in consideration. A detailed case history and third party anamnesis in which driving errors, uncertainties in road traffic, near misses, minor damages, major accidents, compensation strategies, and annual mileage are specifically addressed is required. Neuropsychological tests alone, in particular cognitive short tests, cannot fully inform the decision on driving fitness. Due to the possible progression of dementia, examinations in intervals between half a year to 1 year are required.

### Visual disturbances

Detailed requirements for visual acuity are regulated in §12 and Annex 6 of the FeV and Chapter 3.1 of the Evaluation Guidelines for Driving Ability (BGL) [4].

**Table:** 

**Recommendation** In addition to the regulations of Annex 6 of the FeV, examination of the useful field of vision is recommended to assess the compensatory use of saccadic eye movements after visual field loss.	

### Motor impairments

Appendix B of the Evaluation Guidelines for Driving Ability (BGL) [4] present requirements for the case of motor impairment. Often, motor impairments can be compensated by modifying the vehicle (e.g., automatic transmission or changing pedals for left/right foot use only).

**Table:** 

**Recommendation** Limitation or loss of limb function as a result of a central or peripheral nervous system disorder requires regular neurological evaluations. Compensatory options (e.g., modifications of the vehicle) must be checked within the framework of an on-road driving test.	

### Balance disorders

In Chapter 3.10 of the evaluation guidelines for driving ability (BGL) [4], the different forms of imbalance, dizziness, and vertigo are described in detail. Determining the etiology of the symptoms usually requires a multidisciplinary ENT, internal medical, neurological, and/or psychiatric evaluation.

## Evaluation of the risk potential for sudden loss of control in cerebrovascular disease

Sudden onset symptoms while driving are the cause of accidents in about 1.5 per thousand accidents [8]. The most important accident causing illnesses are epilepsies and cardiovascular diseases. Strokes are the cause of 7% of accidents caused by sudden onset symptoms.

In 2005, a European working group published a detailed risk stratification for various constellations of seizure events [9], the results of which were incorporated into the 2009 edition of the Evaluation Guidelines for Driving Ability (BGL) [4].

Patients with cerebrovascular diseases carry an increased risk of further cerebrovascular diseases, cardiovascular diseases and death. This is similar to the risks associated with heart attacks.

The German Society of Cardiology [10, 11] bases its assessment on the risk stratification developed by the Canadian Cardiovascular Society [12] for cardiac events associated with impaired consciousness, which were adopted in 2016 in Chapter 3.4 Cardiovascular Diseases of the Evaluation Guidelines for Driving Ability (BGL) [4].

### General hazard risk from motor vehicles

The hazard risk of each vehicle group was calculated using the data of the Federal Statistical Office [13] and is shown in Table 1.

**Table 1:** Risk of accidents with damage to persons by main perpetrators

Group 1	Vehicle population	Accidents involving personal injury	Risk per vehicle
Passenger cars	45,803,560	287,710	0.0063
Trucks up to 3.5 tons	2,383,394	12,865	0.0054
Passenger cars plus LKW bis 3.5 tons	48,186,954	300,575	0.0062
Group 2	Vehicle population	Accidents with personal injury	Risk per vehicle
Busses	78,949	3503	0.044
Trucks > 3.5 tons	528,449	5904	0.011
Busses + trucks > 3.5 tons	607,398	9407	0.0155

The risk of accidents with damage to persons for passenger cars + trucks up to 3.5 tons (group 1 without motorcycles) amounts to 40% of that for buses + trucks over 3.5 tons (group 2). The threefold higher risk for busses compared with trucks > 3.5 tons has thus far not been taken into account by the EU Driving Licence Directive [2], the German Driving Permission Act (FeV) [3], or the Evaluation Guidelines for Driving Ability (BGL) [4].

### Assessment of the risk for a loss of control while driving due to a further stroke or cardiovascular event (SCI)

In the current German Evaluation Guidelines for Driving Ability (BGL) [4], this risk is addressed in Chapter 3.9.4 only in the context of transitory ischemic attacks. Apart from the fact that there is also a risk of a further stroke after manifest brain infarctions and that the disturbance of consciousness highlighted as a characteristic of TIA occurs only in 6% of cases (loss of consciousness in < 1% of cases) [14], the risk of a new stroke or cardiovascular event is only a necessary, but not sufficient parameter for determining the level of risk; one also needs to take into account further factors such as amount of time spent driving and others.

Only a single Japanese study is available [15] assessing the risk for a sudden loss of control due to a stroke while driving. Among 2145 stroke patients admitted to an emergency hospital (1301 ischemic insults, 585 cerebral hemorrhages, 259 subarachnoid hemorrhages), 85 (4%) suffered the stroke at the wheel, with a vehicular accident occurring in 14 cases (16% of strokes at the wheel, 0.7% of all stroke patients). Taking into account that individuals deceased at the accident site were not included in this hospital-based examination, and that stroke patients also carry an increased risk of heart attacks, we approximate that about 5% of strokes or cardiovascular events occur at the wheel.

### Risk of damage to persons from an accident resulting from a sudden loss of control (ac)

Several studies are available [16–25] addressing the topic of how frequently damage to persons occurs in accidents that were caused by a sudden loss of control at the wheel.

In these studies a total of 58 damages to persons were reported out of 685 accidents caused by sudden loss of control at the wheel. The probability of damage to persons due to a sudden loss of control at the wheel was thus approximately 8.5%, with the majority of injuries incurred (about 75%) being minor. Our working group proposes 9% for the calculation of Ac according to the Risk of Harm Formula.

The probability of fatal injury to others due to a sudden loss of control at the wheel is 0.8%.

### Assessment of the risk potential after stroke according to the risk of harm formula adapted for the traffic conditions in Germany

This risk assessment is based on the original Risk of Harm Formula developed by the Canadian Cardiovascular Society [12] and the German Society for Cardiology [10] with adaptations to German traffic conditions.

The Risk of Harm Formula is: RH = TD x V x SCI x Ac.

TD = time spent at the wheel per year (25% for professional drivers)

V = Hazard potential of the vehicle (truck = 100%, passenger car = 40%)

SCI = risk of sudden loss of control during driving (5% of the recurrence rate)

Ac = probability of damage to persons due to accident with sudden loss of control (9%)

For a group 2 driver with a relapse risk of 10% per year, the following assessment results:

TD = 25% (time spent at tax per year)

V = 100% (for truck/bus)

SCI = 5% of the recurrence rate per year

Ac = 9%

$$ \mathrm{RH}=0,25\times 1\times \mathrm{0,005}\times 0,09=0,00011 $$

This disease-related risk is 1% of the general risk of trucks > 3.5 tons of 0.011 (group 2) documented in Table 1 and must be added to it. The tolerable SCI for taxi drivers increases with the lower risk of damage to persons of passenger cars (V = 40%). In group 1, shorter travel times reduce the risk.

**Fig. 1:**
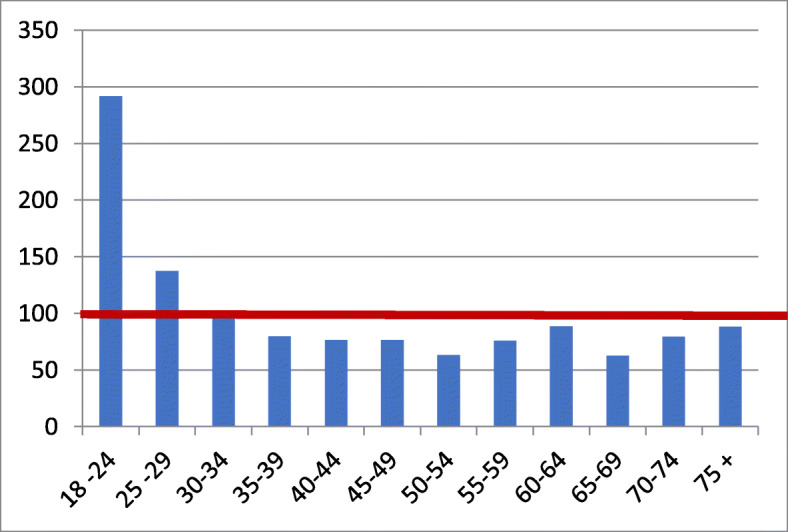
Relative hazard risk in % of mean (mean = 100%). Perpetrators of passenger car accidents with damage to persons per 100,000 driver’s license holders in each age group and per 1000 driving kilometres per year in Germany

In most countries, up to a 1 to 2% additional disease-related risk is considered compatible with motor fitness. This assertion is based, among other things, on the much wider spread of accident rates between different age groups. Figure 1 shows the distribution of main causes of accidents with personal injury per 100,000 driver’s license holders in each age group and per 1000 driving kilometers per year in Germany. We thank the authors of [26] for the kind permission to use their relevant data.

## Prognosis prediction after different types of stroke

### Transitory ischemic attacks (TIA)

The short-term risk of suffering a cerebral infarction after a TIA is 3–10% within 2 days, more than 5% within 7 days, and 9–17% by the 90th day [24]. After that, the risk drops significantly.

Various scoring systems have been developed to predict the risk of stroke. Today, the ABCD2 score is recommended clinically for risk stratification [27]. The advantage of this score lies in its simplicity and the absence of additional technical examinations [28]. Its level of safety in the hands of both neurologists and non-specialists is very high and its risk prediction varies only slightly [29].

The new TIA Registry Study [30] provides useful data for the 1-year risk of all vascular complications:

- TIA 7.1%
- Brain infarct 5.1%
- Death 1.8%
- Acute coronary syndrome 1.1%
- Intracerebral hemorrhage 0.4%

In total the one-year risk after TIA to suffer a serious event that could potentially affect driving ability is less than 15%. TIA patients with macroangiopathy and ABCD2 score of 6 or 7 were particularly at risk. The greatest portion of the risk was spread over the first 3 months, which makes waiting periods necessary. In general, the TIA Registry data indicates a risk reduction compared to older data.

The assessment of the risk of recurrence after TIA and mild cerebral infarction in the Essen database [31] is based on cardiovascular risk factors.

### Brain infarction

**Fig. 2:**
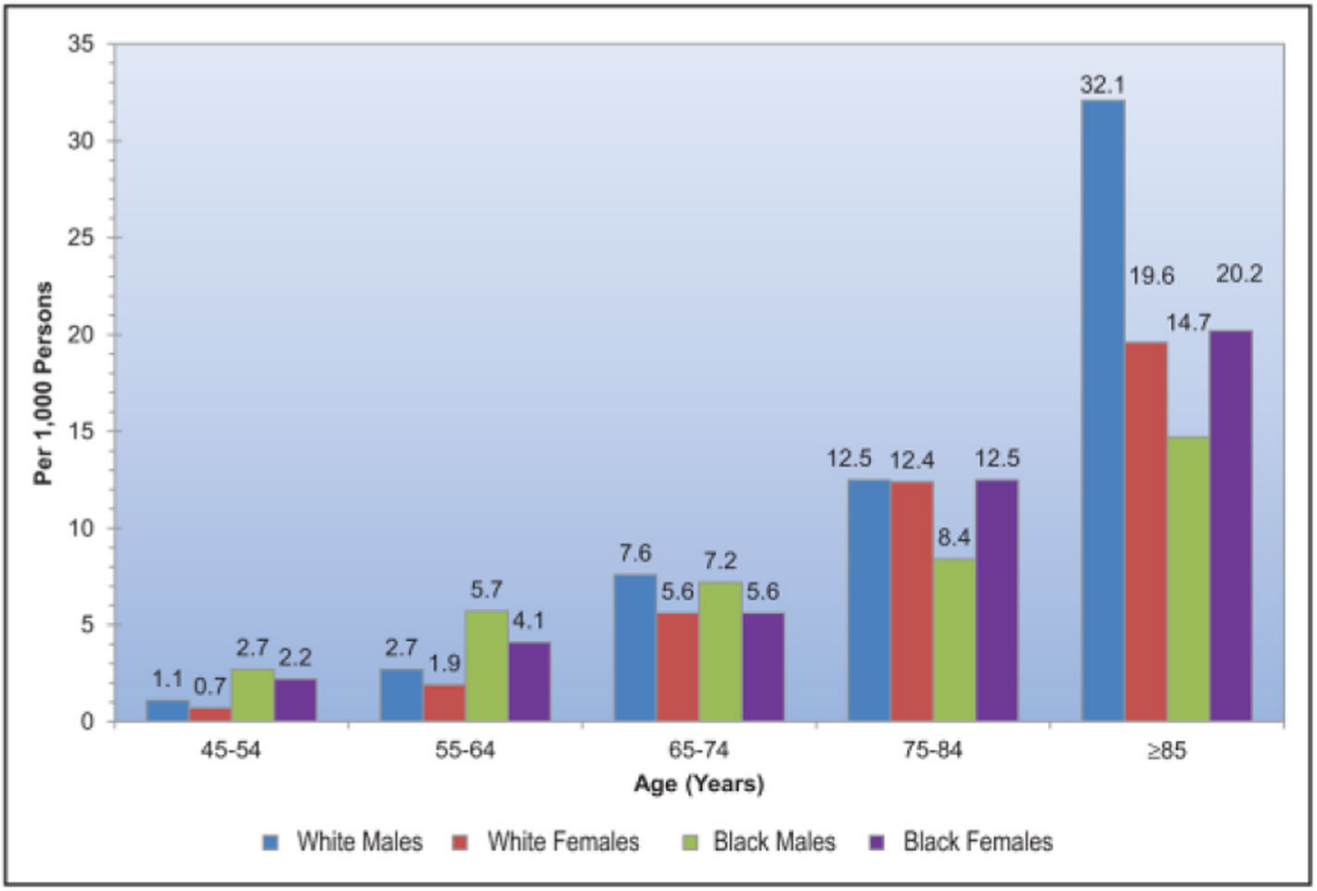
Annual rate of first cerebral infarction by age, sex, and race [32]. Rates for black men and women 45 to 54 years of age and for black men ≥75 years of age are considered unreliable. (Chart 14–3, [32])

The risk of stroke in the general population depends on age and gender [32] (Fig. 2).

This risk level difference must be related to the relative risk of recurrence after a cerebral infarction between genders and age groups. Unfortunately, there is no corresponding comparison table available for the situation after cerebral infarction.

The recurrence rate of secondary events varies from 1 to 4% in the first 30 days, from 6 to 13% in the first year, and from 5 to 8% per year for the next 2–5 years. After 5 years one can assume a 19–42% risk probability for a new stroke event [33–35]. The highest rates of recurrent strokes are reported for macroangiopathic strokes [36].

- 7.9% recurrences for macroangiopathic infarcts
- 6.5% for cardioembolic infarcts
- 6.5% for microangiopathic infarcts.

Seventy percent of recurrences have the same TOAST subtype [37] as the first stroke.

The annual stroke rates in the control arms of clinical trials [38] have continued to decrease over the decades. According to [39], 12 secondary prevention studies found rates of 8.9% per year in the control group and 7.9% per year in the verum groups. The effect of the secondary preventive strategy, with an absolute risk reduction of 1% per year and a relative risk reduction of 12%, can be described as moderate.

The most important publication on brain infarction recurrence is that of the committee of the American Heart Association (AHA) [32]. This publication summarizes the main prior studies and found that in the first year after cerebral infarctions there is an 8% risk of recurrence and a 4% risk of TIAs, death, myocardial infarction, and cerebral hemorrhage. Compared to the general population, this amounts to an average risk increase by a factor of 10.

### Intracerebral hemorrhage

Spontaneous intracerebral hemorrhage (ICH) without a direct source of bleeding (such as an aneurysm or arteriovenous malformation) is predominantly caused by two disease entities: hypertensive cerebral microangiopathy and cerebral amyloid angiopathy [40]. Both variants differ in terms of localization and recurrence rates. Hypertensive degenerative microangiopathy is primarily localized in the areas of basal ganglia and white matter, and cortical areas are largely omitted. In contrast amyloid angiopathy occurs predominantly in the cortex and prefers the parietooccipital region [41].

In 505 patients with ICH in the lobar region, i.e. suspected amyloid angiopathies, 102 ICH recurrences occurred (20,2%), whereas in 640 patients with basal ganglia ICH, i.e. presumably due to hypertensive microangiopathy, only 44 ICH recurrences occurred (6,9%) [40]. Both types profit from blood pressure control. In lobar ICH the event rate was 84/1000 patient years with insufficient blood pressure control versus 49/1000 patient years with sufficient blood pressure control. Basal ganglia ICHs showed 52/1000 patient years with insufficient versus 27/1000 patient years with sufficient blood pressure control. If the primary incidence of intracerebral hemorrhage [42] in Europe is calculated at approx. 32/100,000, there is an increase in risk by a factor of 100 after an ICH.

In a meta-analysis of 10 studies [43] with a total of 1306 patients, the relationship between asymptomatic microhemorrhage in MRI and ICH relapse was investigated. The annual risk of recurrence of ICH was 7.4% for amyloid angiopathy (95% CI- 3.2- 12.6%) compared to 1.1% (95% CI- 0.5- 1.7%) for non-amyloid angiopathy associated ICH. Multiple MRI microhemorrhages increased the risk of recurrence by a factor of 3.1 (95% CI- 1.4- 6.8) for 2–4 microhemorrhages, by 4.3 (95% CI- 1.8- 10.3) for 5–10 microhemorrhages and by 3.4 (95% CI- 1.4- 8.3) for more than 10 microhemorrhages.

### Subarachnoid hemorrhages

In SAH, a distinction must be made between a number of different categories: those with proof of aneurysm, those without proof of aneurysm, convexity SAHs and those with other vascular malformations (AVM, etc.) [44].

Non aneurysmatic perimesencephalic SAH has an extremely low risk of recurrence [45], which is negligible in practice.

Convexity SAHs are most often a consequence of reversible vasoconstriction syndrome (RCVS) in people under 70 years of age and a consequence of amyloid angiopathy in patients over 70 years of age [46]. The risk of recurrence is low.

After an aneurysmal subarachnoid hemorrhage with the aneurysm being eliminated (via clipping or coiling) the risk of recurrence is as high as in the general population [44, 47]. The rate of re-rupture depends on the degree to which the aneurysm is eliminated, but about 90% of re-ruptures occur within the first month after treatment. With an occlusion of 70–90% the risk of re-rupture within a 4 year period is 5.9%, and with less than 70% occlusion the risk of re-rupture within 4 years is 17.6% [48].

The situation is significantly different with bleeding aneurysms that are not sealed off. About 65% of these patients die within the first year [49].

12–20% of acute subarachnoid hemorrhages are immediately fatal, i.e. before medical intervention is possible [50, 51]. This high percentage combined with the typically high intensity of the initial pain make it likely that a sudden loss of control is more frequent in this type of stroke than in any other.

### Non-ruptured aneurysms

The risk of rupture with SAH of a previously unruptured aneurysm is relatively low at 0.8–1.3% per year [44]. But this low risk is about 100 times higher than the risk in the general population of about 1 in 10,000 per year.

**Fig. 3:**
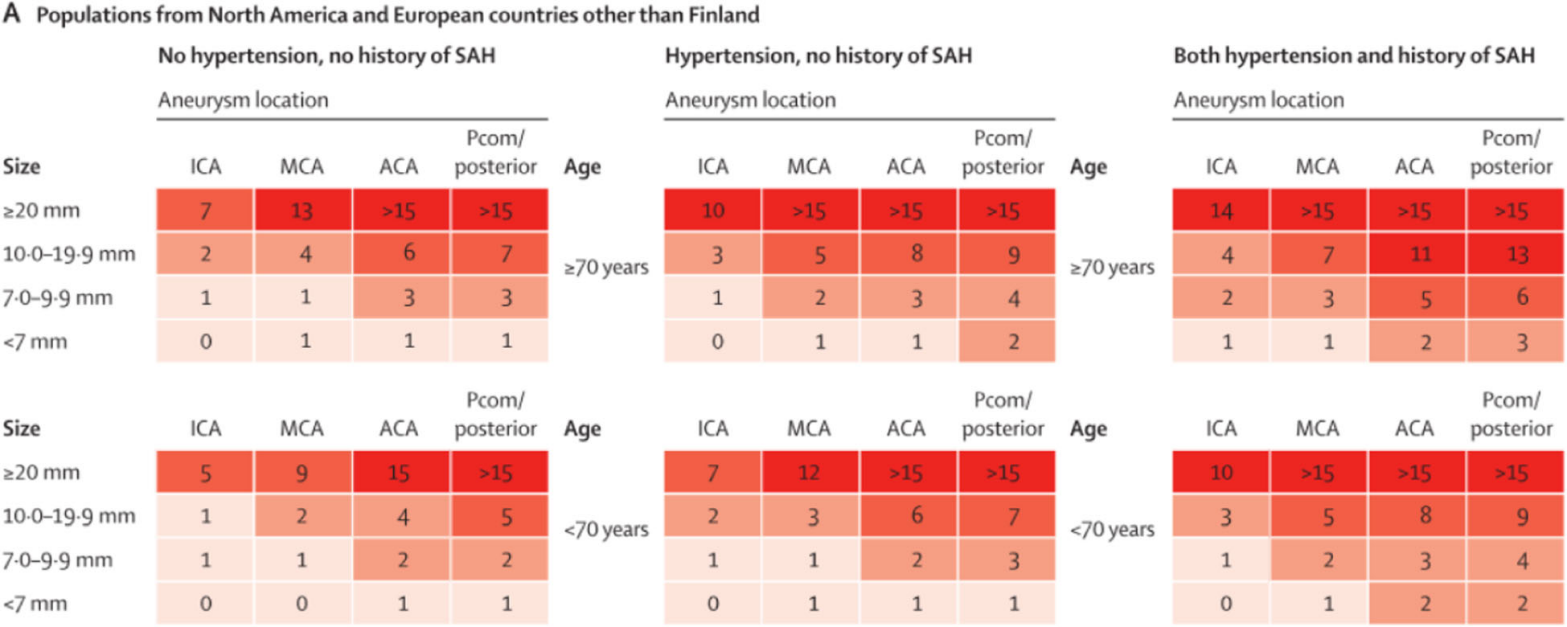
Risk prediction chart for aneurysm rupture (Fig. 2, Part A from [52]). The number in each cell refers to the predicted risk (%) for aneurysm rupture within the next 5 years. ICA = internal carotid artery, MCA = middle cerebral artery, ACA = anterior cerebral artery (including anterior cerebral artery, anterior communicating artery and pericallosal artery), Pcom = posterior communicating artery (including the vertebral artery, basilar artery, cerebellar arteries, and posterior cerebral artery), SAH = subarachnoid hemorrhage

The risk of rupture ranges from 0.25% per year in persons without vascular risk factors and an aneurysm below 7 mm diameter to 15% per year in persons with vascular risk factors and large aneurysms [52]. For further details see the PHASES score, in which high-risk groups are clearly identified (Fig. 3).

### Arteriovenous malformations (AVM)

Patients with AVM (arteriovenous malformations) suffer from congenital malformations of the capillary vascular tract, which allow direct short circuits between arterial vessels and venous outflows via a so-called nidus bypassing the capillary bed [53]. They are endangered by the development of epilepsy and by bleeding events. It is not uncommon for AVM to be detected in the asymptomatic stage by MRT imaging. Their natural course and the long term results of therapeutic measures such as embolisation, radiosurgery or surgery are currently the subject of a number of scientific studies.

The mean annual bleeding risk is estimated to be 3% and ranges between 1 and 33%. The risk increases if:

- a bleeding has occurred already
- the location of the AVM is deep in the brain or brain stem
- the venous drainage is via deep veins

If none of these three factors is present, the annual risk of a neurological event is less than 1%, with one factor the risk is between 3 and 5%, with two factors the risk is between 8 and 15% and with three factors the risk increases to more than 30%.

According to [53], the risk of bleeding is highest in the first 5 years after diagnosis and then decreases significantly.

The ARUBA study showed significantly lower spontaneous bleeding rates in unruptured AVM than previously assumed [54]. In 223 patients, the primary endpoint was met in 10.1% of conservatively treated and 30.7% of interventionally (ie, neurosurgery, embolisation, or stereotactic radiotherapy, alone or in combination) treated patients after a mean follow up of 33,3 months. Interestingly, there were no functional differences between the criteria relevant for the indication of surgery [55] with respect to the prognosis of the patients [56].

### Intracerebral cavernomas

An excellent meta-analysis from 2016 included 25 studies with 90–1295 patients each [57]. It was found that an incidental cavernoma has an annual bleeding rate of 0.3% (95% CI 0.1–0.5%) in the non-brain stem area and of 2.8% (95% CI 2.5–3.3%) in the brain stem area. However, the rebleeding rate after cavernoma bleeding was significantly higher with 6.3% (95% CI 3–13.2%) in the non-brain stem area and 32.3% (95% CI 19.8–52.7%) in the brain stem area. The rate of post-operative bleeding was highest in the first 2 years and occurred on average after 10.5 months. The mortality rate was 2.2%.

### Arteriovenous (AV) fistulas

AV fistulas are rare cerebrovascular diseases that account for only 10–15% of all cerebrovascular malformations [58]. The cause is usually a clinically proven or a silent sinus vein thrombosis with secondary recanalization inducing neoangiogenesis. Accordingly, AV fistulas occur predominantly in patients with primary or secondary coagulation disorders, such as Factor V Leiden mutations, who are at increased risk of sinus vein thrombosis. However, it has been postulated that fistulas could occur spontaneously and trigger secondary sinus vein thrombosis. The rates of non-bleeding neurological deficits (NHND), bleeding and death vary depending on the population investigated and on the drainage type of the fistula. For example, bleeding rates of 35%, NHND of 30% and mortality of 45% were reported in untreated AV fistulas with cortical drainage over a period of 4.3 years [59]. It is important to keep in mind that AV-fistulas can change significantly over time; for example, a harmless fistula without cortical drainage can turn into a dangerous fistula with a pronounced cortical outflow if it increases in size.

The best classifications of AV fistulas are those of Borden et al. [60] and Cognard et al. [61], which classify types according to drainage paths or flow parameters.

The higher the class, the higher the risk of complications. Since this risk is additionally increased in symptomatic AV fistulas, i.e. those after bleeding or with neurological symptoms, it has been proposed to extend the classification system by including asymptomatic (a) and symptomatic (s) [62].

Asymptomatic fistulas of type Borden 2 and 3 or Cognard 2b, 2a + b, 3, 4 and 5 have an annual ICB risk of only 1.4–1.5%.

Therapeutic decisions (transvenous embolization, transarterial embolization, surgical occlusion or radiosurgery) should be made in consultation with a neurovascular case conference and respect to the patient’s expectations.

### Cerebral venous or sinus thrombosis

Cerebral sinus and venous thromboses are rare diseases that usually occur on the basis of predisposing, coagulation-promoting situations such as exsiccation, congenital coagulation defects, pregnancy, or puerperium [63]. Restrictions in fitness to drive are mainly caused by neurological deficits or by accompanying epilepsy.

Recurrences do not play a very important role in the disease and are usually caused by coagulation disorders [64]. Over 39 months, 6% of patients experienced long-term cerebral venous and sinus thrombosis and another 6% experienced venous thrombosis and bleeding complications [62]. 12% within 39 months would roughly correspond to approx. 4% of recurrent illnesses per year. A large part of this does not occur acutely, nor is it associated with a restriction of driving ability.

The recurrence risk in sufficiently anticoagulated patients is, upon completion of the dosing phase, not significantly increased.

## Assessment of driving ability in specific cerebrovascular diseases

The assessment of the driving ability of patients with cerebrovascular disease requires a specified diagnosis and should be carried out after completion of the primary treatment. In addition to the type and extent of existing impairments, an assessment must take into account the disease-specific prognosis indices and therapy options. The driver’s safety awareness, therapy compliance and coping strategies should also be taken into consideration. An interdisciplinary assessment is often advisable.

Due to the possible risk of progression, regular follow-up examinations are necessary every 1 to 2 years.

The following assessment recommendations are based on the assumption of an additional disease-specific risk of 1% (calculated according to the modified Risk of Harm Formula) above the vehicle-specific risk of accidents with damage to persons injury.

The recommendations are intended to provide expert guidance for the indispensable individual assessment. Further guidelines for diseases mentioned in the German Evaluation Guidelines for Driving Ability (BGL) [4], e.g. vision, cardiac arrhythmia, arterial hypertension, diabetes mellitus, and epilepsy, must also be taken into consideration. The waiting periods indicated for individual diseases represent minimum values. Special risk constellations must be evaluated separately and may prohibit driving fitness in individual cases.

**Table:** 

Driving ability in cerebrovascular disease		
Transitory ischemic attacks (TIA)	Group 1	Group 2
Low risk profile, cause treated	Yes	Yes
Waiting period	1 month	3 months
High risk profile (ABCD2 > 6)		
Waiting period	3 month	6 months
Intracranial stenoses and occlusions of large cerebral arteries	Yes	No
Waiting period	6 months	_
Extracranial stenosis and occlusion s. brain infarcts with carotid stenosis		
Brain infarcts	Group 1	Group 2
Intracranial stenoses and occlusions of large cerebral arteries	Yes	No
Waiting period	6 months	–
Severe carotid stenosis after successful desobliteration	Yes	Yes
Waiting period	1 month	3 months
Severe carotid stenosis, conservatively treated	Yes	Yes
Waiting period	3 months	6 months
Unknown cause / low risk profile	Yes	Yes
Waiting period	1 month	3 months
Unknown cause / high risk profile	Yes	Yes
Waiting period	3 month	6 months
Dissection of the large brain-supplying arteries	Yes	Yes
Waiting period	3 month	6 months
Cardio-embolic CHA2DS2-VASC up to 5, anticoagulated	Yes	Yes
Waiting period	1 month	3 months
Cardio-embolic CHA2DS2-VASC up to 5, not anticoagulated	Yes	No
Waiting period	6 month	–
Cardio-embolic CHA2DS2-VASC > 5, anticoagulated	Yes	Yes
Waiting period	1 month	3 months
Cardio-embolic CHA2DS2-VASC > 5, not anticoagulated	No	No
Waiting period	–	–
Microangiopathic	Yes	Yes
Waiting period	1 month	3 months
Cerebral vasculitis	Group 1	Group 2
Giant cell arteritis, untreated	No	No
Waiting period	–	–
Giant cell arteritis, treated ESR and CRP normalised for 4 weeks	Yes	Yes
Waiting period	None	None
Other cerebral vasculitis, if under treatment controlled	Yes	Yes
Waiting period depending on the prognosis of the disease	3–12 month	6–12 months
Brain hemorrhage	Group 1	Group 2
Amyloid angiopathy / symptomatic bleeding + more than 5 asymptomatic bleedings or superficial siderosis	No	No
Waiting period	–	–
Single hypertensive bleeding / blood pressure within normal range	Yes	Yes
Waiting period	1 month	3 months
Single hypertensive bleeding / blood pressure not within normal range	No	No
Waiting period	–	–
More than 2 hypertensive bleedings within 5 years	No	No
Waiting period	–	–
Subarachnoid hemorrhage	Group 1	Group 2
Non-aneurysmatic perimesencephalic/prepontine/convexity	Yes	Yes
Waiting period	2 weeks	2 weeks
Aneurysm occluded	Yes	Yes
Waiting period	1 month	1 month
Aneurysm not occluded	No	No
Waiting period	–	–
Asymptomatic, unruptured aneurysm	Group 1	Group 2
Bleeding risk up to 4%/year	Yes	Yes
Waiting period	None	None
Bleeding risk > 4%/year	No	No
Waiting period	–	–
Aneurysm occluded	Yes	Yes
Waiting period	1 month	1 month
Arterio-venous malformations	Group 1	Group 2
Not ruptured, without deep or brainstem involvement and without deep venous drainage (accidental finding)	Yes	Yes
Waiting period	None	None
Ruptured, untreated	Yes	Yes
Waiting period	3 years	5 years
Ruptured, completely removed	Yes	Yes
Waiting period	None	None
Ruptured, treatment not yet completed	Yes	Yes
Waiting period	3 years	5 years
Cavernoma	Group 1	Group 2
Accidental finding, no bleeding, not located in the brain stem	Yes	Yes
Waiting period	None	None
Accidental finding, no bleeding, located in the brain stem	Yes	No
Waiting period	None	–
Surgically removed	Yes	Yes
Waiting period	3 months	3 months
Bled, not removed, not located in the brain stem	Yes	Yes
Waiting period	2 years	2 years
Bled, not removed, located in the brain stem	Yes	No
Waiting period	2 years	–
Arterio-venous fistulae	Group 1	Group 2
Asymptomatic	Yes	Yes
Waiting period	None	None
Symptomatic, high risk (type Boden 2 and 3, Cognard 2b-5)	No	No
Waiting period	–	–
Completely removed	Yes	Yes
Waiting period	1 week	1 week
Cerebral venous or sinus thrombosis	Group 1	Group 2
Without congenital coagulation defects	Yes	Yes
Waiting period	None	None
With congenital coagulation defects, anticoagulated	Yes	Yes
Waiting period	1 month	1 month

## Data Availability

Literature search.

## Abbreviations

ABCD2: Age, blood pressure, clinical features, Diabetes mell
AHA: American Heart Association
AV: Arteriovenous
AVM: Arteriovenous malformation
BASt: Bundesanstalt für Straßenwesen (Federal Highway Research Institute)
BGL: Begutachtungsleitlinien zur Kraftfahreignung (Evaluation guidelines for driving ability)
CRP: C-reactive protein
DGN: Deutsche Gesellschaft für Neurologie
DGNB: Deutsche Gesellschaft für neurowissenschaftliche Begutachtung
DGNC: Deutsche Gesellschaft für Neurochirurgie
DGNR: Deutsche Gesellschaft für Neurorehabilitation
DSG: Deutsche Schlaganfallgesellschaft
ESR: Erythrocyte sedimentaion rate
EU: European Union
FeV: Fahrerlaubnisverordnung (German Driving Permission Act)
GCNKSS: Greater Cincinnati/Northern Kentucky Stroke Study
GNP: Gesellschaft für Neuropsychologie
ICH: Intracerebral hemorrhage
MRI: Magnetic resonance imaging
mRS: Modified Rankin Scale
NHND: Nonhemorrhagic neurological deficits
SAH: Subarachnoid hemorrhage
StVG: Straßenverkehrsgesetz (German Road Traffic Act)
TIA: Transient ischemic attack
TOAST: Trial of Org 10,172 in Acute Stroke Treatment

## References

[1] Bundesministerium-der-Justiz-und-für-Verbraucherschutz (2018). *Straßenverkehrsgesetz (StVG)*.

[2] EU-Commission (2006). *Richtlinie 2006/126/EG des Europäischen Parlaments und des rates vom 20. Dezember 2006 über den Führerschein (Neufassung)*.

[3] Bundesministerium-für-Justiz-und-Verbraucherschutz-BMJV (2016). *Verordnung über die Zulassung von Personen zum Straßenverkehr (Fahrerlaubnis-Verordnung - FeV)*.

[4] Gräcmann N, Albrecht M, Bundesanstalt-für-Straßenwesen (2018). *Begutachtungsleitlinien zur Kraftfahreignung*.

[5] Golper LA, Rau MT, Marshall RC (1980). Aphasic adults and their decisions on driving: An evaluation. Archives of Physical Medicine and Rehabilitation.

[6] Hartje W (1991). Driving ability of aphasic and non-aphasic brain-damaged patients. Neuropsychological Rehabilitation.

[7] Deuschl G, Maier W (2016). *Demenzen*, in *AWMF-Leitlinien*.

[8] Petch MC (1998). Driving and heart disease. European Heart Journal.

[9] Second-European-Working-Group-on-Epilepsy-and-Driving (2005). *Epilepsy and driving in Europe*.

[10] Klein H (2010). Fahreignung bei kardiovaskulären Erkrankungen. Kardiologe.

[11] Klein HH, Sechtem U, Trappe HJ (2017). Fahreignung bei kardiovaskulären Erkrankungen. Deutsches Ärzteblatt International.

[12] Simpson C (2004). Assessment of the cardiac patient for fitness to drive: Drive subgroup executive summary. The Canadian Journal of Cardiology.

[13] Statistisches-Bundesamt, Verkehrsunfälle (2017). Fachserie 8.

[14] Compter A (2014). Is the long-term prognosis of transient ischemic attack or minor ischemic stroke affected by the occurrence of nonfocal symptoms?. Stroke.

[15] Inamasu J (2018). Stroke while driving: Frequency and association with automobile accidents. International Journal of Stroke.

[16] Myerburg RJ, Davis JH (1964). The medical ecology of public safety. I. Sudden death due to coronary heart disease. American Heart Journal.

[17] Herner B, Smedby B, Ysander L (1966). Sudden illness as a cause of motor-vehicle accidents. British Journal of Industrial Medicine.

[18] West I (1968). Natural death at the wheel. JAMA.

[19] Cheng LH, Whittington RM (1998). Natural deaths while driving: Would screening for risk be ethically justified?. Journal of Medical Ethics.

[20] Ostrom M, Eriksson A (1987). Natural death while driving. Journal of Forensic Sciences.

[21] Osawa M (1998). Sudden natural death in driving: Case studies in the western area of Kanagawa. Nihon Hōigaku Zasshi.

[22] Buttner A, Heimpel M, Eisenmenger W (1999). Sudden natural death ‘at the wheel’: A retrospective study over a 15-year time period (1982-1996). Forensic Science International.

[23] Berg AT (2000). Driving in adults with refractory localization-related epilepsy*. Multi-Center Study of Epilepsy Surgery*. Neurology.

[24] Parsons M (1986). Fits and other causes of loss of consciousness while driving. The Quarterly Journal of Medicine.

[25] Christian MS (1988). Incidence and implications of natural deaths of road users. BMJ.

[26] Casutt G, Jäncke L (2015). Straßenverkehrsunfälle im Ländervergleich: Unterschiedliche Unfallrate bei Senioren zwischen Deutschland und der Schweiz. Z. f. Verkehrssicherheit.

[27] Johnston SC (2007). Validation and refinement of scores to predict very early stroke risk after transient ischaemic attack. Lancet.

[28] Wolf ME, Held VE, Hennerici MG (2014). Risk scores for transient ischemic attack. Frontiers of Neurology and Neuroscience.

[29] Sanders LM (2012). Performance of the ABCD2 score for stroke risk post TIA: Meta-analysis and probability modeling. Neurology.

[30] Amarenco P (2016). One-year risk of stroke after transient ischemic attack or minor stroke. The New England Journal of Medicine.

[31] Weimar C (2009). The Essen stroke risk score predicts recurrent cardiovascular events: A validation within the REduction of Atherothrombosis for continued health (REACH) registry. Stroke.

[32] Benjamin EJ (2017). Heart disease and stroke Statistics-2017 update: A report from the American Heart Association. Circulation.

[33] Rundeck T, Sacco R, Mohr P (2011). Prognosis after stroke. *Stroke pathophysiology, diagnosis, and management*.

[34] Lee BI (2001). Yonsei stroke registry. Analysis of 1,000 patients with acute cerebral infarctions. Cerebrovascular Diseases.

[35] Hillen T (2003). Cause of stroke recurrence is multifactorial: Patterns, risk factors, and outcomes of stroke recurrence in the South London stroke register. Stroke.

[36] Jones SB (2013). Poststroke outcomes vary by pathogenic stroke subtype in the atherosclerosis risk in communities study. Stroke.

[37] Adams HP (1993). Classification of subtype of acute ischemic stroke. Definitions for use in a multicenter clinical trial. TOAST. Trial of org 10172 in acute stroke treatment. Stroke.

[38] Hong KS (2011). Declining stroke and vascular event recurrence rates in secondary prevention trials over the past 50 years and consequences for current trial design. Circulation.

[39] Hankey GJ (2014). Secondary stroke prevention. Lancet Neurology.

[40] Biffi A (2015). Association between blood pressure control and risk of recurrent intracerebral hemorrhage. JAMA.

[41] Banerjee G (2017). The increasing impact of cerebral amyloid angiopathy: Essential new insights for clinical practice. Journal of Neurology, Neurosurgery, and Psychiatry.

[42] Sacco S (2009). Incidence and 10-year survival of intracerebral hemorrhage in a population-based registry. Stroke.

[43] Charidimou A (2017). Brain hemorrhage recurrence, small vessel disease type, and cerebral microbleeds: A meta-analysis. Neurology.

[44] Steiner T (2013). European stroke organization guidelines for the management of intracranial aneurysms and subarachnoid haemorrhage. Cerebrovascular Diseases.

[45] Hong Y (2017). Recurrent Perimesencephalic nonaneurysmal subarachnoid hemorrhage: Case report and review of the literature. World Neurosurgery.

[46] Khurram A, Kleinig T, Leyden J (2014). Clinical associations and causes of convexity subarachnoid hemorrhage. Stroke.

[47] Lawton MT, Vates GE (2017). Subarachnoid hemorrhage. The New England Journal of Medicine.

[48] Johnston SC (2008). Predictors of rehemorrhage after treatment of ruptured intracranial aneurysms: The cerebral aneurysm Rerupture after treatment (CARAT) study. Stroke.

[49] Korja M (2017). Natural history of ruptured but untreated intracranial aneurysms. Stroke.

[50] Schievink WI (1995). Sudden death from aneurysmal subarachnoid hemorrhage. Neurology.

[51] Huang J, van Gelder JM (2002). The probability of sudden death from rupture of intracranial aneurysms: A meta-analysis. Neurosurgery.

[52] Greving JP (2014). Development of the PHASES score for prediction of risk of rupture of intracranial aneurysms: A pooled analysis of six prospective cohort studies. Lancet Neurology.

[53] Solomon RA, Connolly ES (2017). Arteriovenous malformations of the brain. The New England Journal of Medicine.

[54] Mohr JP (2014). Medical management with or without interventional therapy for unruptured brain arteriovenous malformations (ARUBA): A multicentre, non-blinded, randomised trial. Lancet.

[55] Spetzler RF, Martin NA (2008). *A proposed grading system for arteriovenous malformations.* 1986. J Neurosurg.

[56] Mohr JP (2017). Functional impairments for outcomes in a randomized trial of unruptured brain AVMs. Neurology.

[57] Taslimi S (2016). Natural history of cavernous malformation: Systematic review and meta-analysis of 25 studies. Neurology.

[58] Miller TR, Gandhi D (2015). Intracranial Dural arteriovenous fistulae: Clinical presentation and management strategies. Stroke.

[59] van Dijk JM (2002). Clinical course of cranial dural arteriovenous fistulas with long-term persistent cortical venous reflux. Stroke.

[60] Borden JA, Wu JK, Shucart WA (1995). A proposed classification for spinal and cranial dural arteriovenous fistulous malformations and implications for treatment. Journal of Neurosurgery.

[61] Cognard C (1995). Cerebral dural arteriovenous fistulas: Clinical and angiographic correlation with a revised classification of venous drainage. Radiology.

[62] Zipfel GJ (2009). Cranial dural arteriovenous fistulas: Modification of angiographic classification scales based on new natural history data. Neurosurgical Focus.

[63] Ferro JM, Canhao P (2014). Cerebral venous sinus thrombosis: Update on diagnosis and management. Current Cardiology Reports.

[64] Hiltunen S (2016). Long-term outcome after cerebral venous thrombosis: Analysis of functional and vocational outcome, residual symptoms, and adverse events in 161 patients. Journal of Neurology.

